# Hormonal Regulation of Plant Growth and Development

**DOI:** 10.1371/journal.pbio.0020311

**Published:** 2004-09-14

**Authors:** William M Gray

## Abstract

Besides environmental factors, plant growth depends upon endogenous signals. Bill Gray examines what these hormonal signals are and how they act to regulate many aspects of growth and development.

## Phytohormones: What Are they?

Plant growth and development involves the integration of many environmental and endogenous signals that, together with the intrinsic genetic program, determine plant form. Fundamental to this process are several growth regulators collectively called the plant hormones or phytohormones. This group includes auxin, cytokinin, the gibberellins (GAs), abscisic acid (ABA), ethylene, the brassinosteroids (BRs), and jasmonic acid (JA), each of which acts at low concentrations to regulate many aspects of plant growth and development.

**Figure 1:**
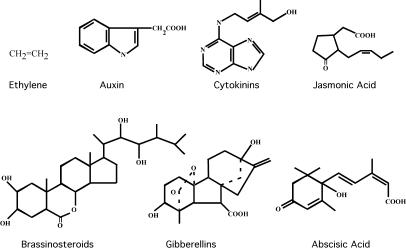
Chemical Structures of the Plant Hormones A partial list of the responses elicited by each hormone is provided below. Ethylene gas promotes fruit ripening, senescence, and responses to pathogens and abiotic stresses. IAA (an auxin) regulates cell division and expansion, vascular differentiation, lateral root development, and apical dominance. Cytokinins are adenine derivatives first identified by their ability to promote cytokinesis. JA is a volatile signal that modulates pollen development and responses to pathogen infection. The BRs regulate cell expansion and photomorphogenesis (light-regulated development). GAs are diterpenoid compounds that promote germination, stem elongation, and the induction of flowering. ABA promotes seed dormancy and is involved in several stress signaling pathways.

With the notable exception of the steroidal hormones of the BR group, plant hormones bear little resemblance to their animal counterparts (Figure 1). Rather, they are relatively simple, small molecules such as ethylene gas and indole-3-acetic acid (IAA), the primary auxin in the majority of plant species. The concept of plant hormones originates from a classical experiment on phototropism, the bending of plants toward light, carried out by Charles Darwin and his son Francis in 1880. The Darwins were able to demonstrate that when oat seedlings were exposed to a lateral light source, a transported signal originating from the plant apex promoted differential cell elongation in the lower parts of the seedling that resulted in it bending toward the light source. This signal was subsequently shown to be IAA, the first known plant hormone.

## What Do They Do?

Virtually every aspect of plant growth and development is under hormonal control to some degree. A single hormone can regulate an amazingly diverse array of cellular and developmental processes, while at the same time multiple hormones often influence a single process. Well-studied examples include the promotion of fruit ripening by ethylene, regulation of the cell cycle by auxin and cytokinin, induction of seed germination and stem elongation by GA, and the maintenance of seed dormancy by ABA. Historically, the effects of each hormone have been defined largely by the application of exogenous hormone. More recently, the isolation of hormone biosynthetic and response mutants has provided powerful new tools for painting a clearer picture of the roles of the various phytohormones in plant growth and development.

## How Do They Work?

Plant biologists have been fascinated by the regulatory capacity of phytohormones since the time of their discovery, and the notion that hormone levels or responses could be manipulated to improve desired plant traits has long been an area of intense interest. Perhaps the best-known example of this is the isolation of dwarf varieties of wheat and rice that led to the “green revolution” in the second half of the 20th century, which is credited with saving millions of people around the globe from starvation. These dwarf varieties have shorter stems than wild-type, making these plants less susceptible to damage by wind and rain. The molecular isolation of these “dwarfing genes” has revealed that they encode components of the GA biosynthesis and response pathways (Peng et al. 1999; Sasaki et al. 2002).

To elucidate the molecular mechanisms underlying phytohormone action, several researchers have utilized the genetically facile model plant Arabidopsis thaliana to isolate mutations that confer altered response to applied hormone. Molecular and biochemical analysis of the gene products defined by these mutations, coupled with expression studies aimed at identifying the downstream target genes that mediate hormonal changes in growth and development, has begun to unlock some of the mysteries behind phytohormone action. While no hormone transduction pathway is completely understood, we now have a rudimentary understanding of many of the molecular events underlying hormone action. Several reviews covering the individual hormone pathways in greater detail have recently been published (Turner et al. 2002; Gomi and Matsuoka 2003; Himmelbach et al. 2003; Kakimoto 2003; Dharmasiri and Estelle 2004; Guo and Ecker 2004; Wang and He 2004).

## Common Themes

**Figure 2:**
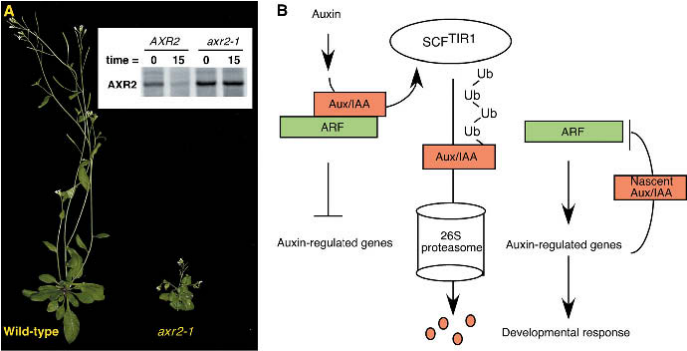
The Ubiquitin-Mediated Proteolysis of Aux/IAA Proteins Regulates Auxin Response (A) Wild-type Arabidopsis thaliana and the *axr2-1* mutant. *axr2-1* is a dominant gain-of-function mutation in an Aux/IAA gene that confers reduced auxin response. The mutant *axr2-1* protein constitutively represses auxin response because it cannot be targeted for proteolysis by the SCF^TIR1^ ubiquitin ligase. The effect of the mutation on AXR2 stability is shown in a pulse-chase experiment (inset). Wild-type and *axr2-1* seedlings were labeled with ^35^S-methionine and AXR2/axr2-1 protein was immunoprecipitated either immediately after the labeling period (t = 0) or following a 15-minute chase with unlabeled methionine (t = 15). (B) A simplified model for auxin response. In the absence of an auxin stimulus, Aux/ IAA proteins inhibit ARF transcriptional activity by forming heterodimers. Auxin perception (by an unknown receptor) targets the Aux/IAA proteins to the SCF^TIR1^ complex, resulting in their ubiquitination and degradation, thereby de-repressing the ARF transcription factors. Among the ARF targets are the *Aux/IAA* genes themselves, which produce nascent Aux/IAA proteins that restore repression upon the pathway in a negative feedback loop.

Regulation by proteolysis has emerged as a resounding theme in plant hormone signaling. The ubiquitin-mediated degradation of key regulatory proteins has been demonstrated, or is at least likely, for all of the phytohormone response pathways (Smalle and Vierstra 2004). In the case of auxin, the response pathway is normally subject to repression by a large family of transcriptional regulators called the Aux/IAA proteins (Figure 2). These proteins dimerize with members of the auxin response factor (ARF) family of transcription factors, thus preventing ARFs from activating auxin-responsive genes (Tiwari et al. 2004). Upon an auxin stimulus, an SCF (SKP1/Cullin/F-box protein) ubiquitin ligase (Deshaies 1999) containing the TIR1 F-box protein ubiquitinates the Aux/IAA proteins, marking them for degradation by the 26S proteasome thereby de-repressing the response pathway (Gray et al. 2001). The hormone promotes the Aux/IAA–TIR1 interaction; however, the molecular mechanisms behind this regulation are unclear. Most yeast and animal SCF substrates must be post-translationally modified, usually by phosphorylation, before they are recognized by their cognate F-box protein. Despite numerous efforts to identify auxin-induced modification of Aux/IAA proteins, no such signal has been discovered, raising the distinct possibility that auxin uses a novel mechanism to regulate SCF–substrate interactions.

Ethylene and cytokinin are both perceived by receptors sharing similarity to bacterial two-component regulators. Common in prokaryotes, but apparently restricted to plants and fungi in eukaryotes, these modular signaling systems involve a membrane-bound receptor containing an intracellular histidine kinase (HK) domain (Wolanin et al. 2002). Ligand binding activates the kinase, resulting in autophosphorylation and initiation of a series of phosphotransfer reactions that culminates with the activation of a response regulator protein that functions as the effector component of the pathway. Cytokinin signaling appears to largely follow this paradigm (Kakimoto 2003). Ethylene response, however, appears more complex (Guo and Ecker 2004).

Ethylene is perceived by a family of five receptors. ETR1 and ERS1 contain a consensus HK domain, however, the HK domains of ETR2, ERS2, and EIN4 are degenerate and lack elements necessary for catalytic activity. This fact, together with studies of “kinase-dead” mutants of *ETR1*, suggests that HK activity is not required for ethylene response. Mutations that abolish ethylene binding in any of the five receptor genes are dominant and confer ethylene insensitivity, indicating that the receptors function as negative regulators of the ethylene pathway.

**Figure 3:**
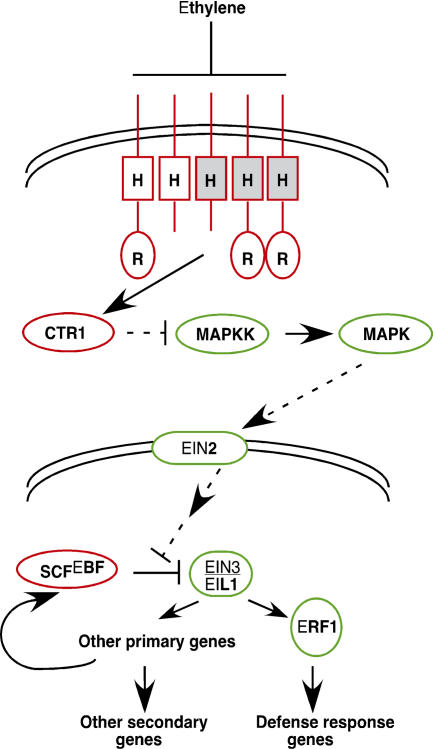
A Model for the Arabidopsis Ethylene Response Pathway Ethylene is perceived by a family of two-component receptors containing a consensus (unshaded) or degenerate (shaded) HK domain (H). Three of the receptors also contain a C-terminal receiver domain (R). The receptors negatively regulate ethylene response together with CTR1 in a complex on the endoplasmic reticulum membrane. Perception results in reduced receptor and CTR1 activities and activation of a MAP kinase kinase, which transmits the signal through the EIN2 membrane protein, ultimately resulting in the activation of a transcriptional cascade in the nucleus. The EIN3 and EIL1 transcription factors regulate primary response genes including *ERF1*, which activates a subset of secondary ethylene-induced genes involved in defense responses. EIN3/EIL1 abundance is regulated in an ethylene-dependent manner by SCF complexes containing F-box proteins encoded by the ethylene-induced genes *EBF1* and *EBF2*. Positive- and negative-acting components of the pathway are indicated in green and red, respectively. Solid lines indicate regulation that is likely to be through direct interactions. Dotted lines indicate speculative interactions based on genetic studies.

Genetic and molecular studies have positioned these receptors upstream of the Raf-like MAP kinase kinase kinase, CTR1, which interacts with the receptors and also acts as a negative regulator (Figure 3). The integral membrane protein, EIN2, and the transcription factors EIN3 and EIL1 are positive regulators of ethylene signaling downstream of CTR1. Current models propose that hormone binding inactivates the receptors, thus resulting in down-regulation of CTR1 activity. Since the identification of CTR1, biologists have speculated that a MAP kinase cascade may be involved. Only recently, however, have putative MAP kinase kinase and MAP kinase components of the ethylene pathway been identified (Chang 2003). Interestingly, these kinases appear to positively regulate ethylene response, suggesting that CTR1 must inhibit their function. If so, this would represent a novel twist on the traditional MAP kinase signaling paradigm. Precisely how the ethylene signal is transduced to the EIN3 and EIL1 transcription factors remains unclear. However, the recent finding that ethylene stabilizes these transcription factors, which are targeted for degradation by an SCF complex in the absence of ethylene, clearly indicates a role for the ubiquitin pathway (Guo and Ecker 2003; Potuschak et al. 2003). One of the known targets for EIN3 is the ERF1 transcription factor, which activates several genes involved in a subset of ethylene responses.

## Signal Integration and Combinatorial Control

Long ago, plant physiologists noted the apparent antagonistic interactions between some of the phytohormones, such as between auxin and cytokinin in the regulation of root–shoot differentiation and between GA and ABA in germination. Other processes are synergistically regulated by multiple hormones. While it has long been obvious that hormones do not function in discrete pathways, but rather exhibit extensive cross-talk and signal integration with each other and with environmental and developmental signaling pathways, the molecular basis for such coordinated regulation has been unclear. Several recent findings have begun to elucidate the molecular details of some of these events.

One example of such signal integration was recently described for the ethylene and JA pathways (Lorenzo et al. 2003). Genetic studies had previously implicated both hormones as important regulators of pathogen defense responses, as well as of the wounding response and other stress-related pathways. Additionally, microarray analysis has identified a large number of genes that are responsive to both hormones. The ERF1 transcription factor was recently found to be an intersection point for these two signaling pathways (Lorenzo et al. 2003). Like ethylene, JA rapidly induces *ERF1* expression, and treatment with both hormones synergistically activates *ERF1*. Induction of *ERF1* by both hormones alone or in combination is dependent upon both signaling pathways, and constitutive overexpression of *ERF1* rescues the defense-response defects of both ethylene- and JA-insensitive mutants. These findings suggest that *ERF1* represents one of the first signaling nodes identified in the complex web of hormonal cross-talk.

The auxin and BR pathways also appear to converge and mutually regulate some developmental processes. Both hormones promote cell expansion, and microarray studies have revealed that as many as 40% of all BR-induced genes are also up-regulated by auxin (Goda et al. 2004; Nemhauser et al. 2004). BR is perceived by the cell surface receptor kinase BRI1 (Wang and He 2004). The SHAGGY/GSK3-type kinase BIN2 acts as a negative regulator of the pathway downstream of the receptor. In the absence of a BR signal, BIN2 phosphorylates the transcription factors BES1 and BZR1, targeting them for proteolysis by the 26S proteasome. Upon a BR stimulus, BIN2 is inactivated, allowing BES1 and BZR1to accumulate in the nucleus, where they are presumably involved in regulating BR-responsive genes.

Using combined genetic, physiological, and genomic approaches, Nemhauser and colleagues (2004) were able to demonstrate that auxin and BR regulate Arabidopsis hypocotyl (embryonic stem) elongation in a synergistic and interdependent fashion. Elevating endogenous auxin levels rendered plants more sensitive to BR application in hypocotyl elongation assays, and this response was dependent upon both the auxin and BR signaling pathways. Genetic studies suggest that the convergence of these two pathways occurs at a late point in hormone signaling, perhaps at the promoters of the many genes responsive to both hormones. In support of this notion, bioinformatic analysis identified distinct sequence elements that were enriched specifically in the promoters of auxin-induced, BR-induced, and auxin/BR-induced genes.

## Many Unanswered Questions

While great strides have been made in recent years in understanding the molecular basis of phytohormone action, many fundamental questions remain. Receptors and other upstream signaling components remain to be identified for the majority of the phytohormones. Equally important are the elucidation of hormonal networks and the integration of these networks with the morphogenetic program, such that our understanding of hormone action can be placed in a developmental context.

## Abbreviations

ABA: abscisic acid
ARF: auxin response factor
BR: brassinosteroid
GA: gibberellin
HK: histidine kinase
IAA: indole-3-acetic acid
JA: jasmonic acid
SCF: SKP1/Cullin/F-box protein
